# Understanding Reactive Oxygen Species in Bone Regeneration: A Glance at Potential Therapeutics and Bioengineering Applications

**DOI:** 10.3389/fbioe.2022.836764

**Published:** 2022-02-07

**Authors:** Aaron J. Sheppard, Ann Marie Barfield, Shane Barton, Yufeng Dong

**Affiliations:** ^1^ Department of Orthopaedic Surgery, Louisiana State University Health Shreveport, Shreveport, LA, United States; ^2^ School of Medicine, Louisiana State University Health Shreveport, Shreveport, LA, United States

**Keywords:** reactive oxygen species, redox signaling, bone healing, skeletal biology, regenerative medicine, tissue engineering, biomaterials

## Abstract

Although the complex mechanism by which skeletal tissue heals has been well described, the role of reactive oxygen species (ROS) in skeletal tissue regeneration is less understood. It has been widely recognized that a high level of ROS is cytotoxic and inhibits normal cellular processes. However, with more recent discoveries, it is evident that ROS also play an important, positive role in skeletal tissue repair, specifically fracture healing. Thus, dampening ROS levels can potentially inhibit normal healing. On the same note, pathologically high levels of ROS cause a sharp decline in osteogenesis and promote nonunion in fracture repair. This delicate balance complicates the efforts of therapeutic and engineering approaches that aim to modulate ROS for improved tissue healing. The physiologic role of ROS is dependent on a multitude of factors, and it is important for future efforts to consider these complexities. This review first discusses how ROS influences vital signaling pathways involved in the fracture healing response, including how they affect angiogenesis and osteogenic differentiation. The latter half glances at the current approaches to control ROS for improved skeletal tissue healing, including medicinal approaches, cellular engineering, and enhanced tissue scaffolds. This review aims to provide a nuanced view of the effects of ROS on bone fracture healing which will inspire novel techniques to optimize the redox environment for skeletal tissue regeneration.

## 1 Introduction

The influx of skeletal biology research over the past 20 years has improved our understanding of how bones develop, remodel, and repair via very complex mechanisms that requires the interaction of cells from different lineages (General 2004; Hadjidakis and Androulakis 2006). These cells (including osteoclasts, osteoblasts, osteocytes, lymphoid cells, neurons, and vascular cells) each respond to a myriad of signaling cascades and external factors to regulate bone homeostasis and repair; thus, any dysfunction of this system can lead to bone pathology or suboptimal repair (Hadjidakis and Androulakis 2006; Jeon et al., 2016; Lopes et al., 2018). Reactive oxygen species (ROS) are increasingly being recognized as a key component of the bone repair paradigm. Are they “good”, or are they “bad”? There is much debate over the role ROS play in the entire bone repair process, as some studies show they are necessary for bone repair and others say they are detrimental to the process. This review provides a general understanding of how ROS interact and influence key regulators in the bone healing process. Given that there are limited studies specifically investigating the role of ROS in bone healing, this first section compiles the existing literature from *in vitro* and pre-clinical studies to suggest their potential role. The second half reviews the existing evidence for using supplements, drugs, and bioengineering techniques to harness (or dampen) ROS levels to improve fracture healing. For better therapeutics and bioengineering approaches to be developed, we need to understand how they influence the oxidative balance during skeletal tissue healing.

### 1.1 Bone Healing After Initial Injury

Typically, the first phase in injury healing involves the disruption of local blood vessels, resulting in inflammatory hematoma formation (Schlundt et al., 2015; Sivaraj and Adams 2016). Blood cells, resident bone macrophages (osteomacs), local mesenchymal stem cells (MSCs), and damaged endothelial cells all contribute to directing inflammatory signals to the damaged site (Lopes et al., 2018). These signals are released in a controlled manner to further recruit osteoprogenitor cells, inflammatory cells, and platelets to the area (Mountziaris and Mikos 2008; Lopes et al., 2018). Peak inflammation occurs 24 h after injury, and the entire inflammatory response is usually complete after 1 week (Mountziaris and Mikos 2008). MSCs respond to signals from the damaged area and are recruited to the site where they can begin the process of forming bone tissue. MSCs first cluster together in the hypoxic, avascular area (Percival and Richtsmeier 2013). In fact, it is believed this zone of slight hypoxia and avascularity is necessary for this first mesenchymal condensation to initiate (M. Yin and Pacifici 2001). These MSCs respond to a variety of signaling molecules, mechanical stresses, and oxygen tension to decide whether to differentiate towards osteoblasts or chondrocytes (Hadjidakis and Androulakis 2006; Percival and Richtsmeier 2013; Bahney et al., 2019). As an oversimplified explanation, the MSCs in the inner mass of the MSC condensation favor chondrogenesis, while MSCs in more close contact with inflammatory signals and blood supply favor osteogenesis (Percival and Richtsmeier 2013; Bahney et al., 2019). As the outer MSCs differentiate and the neovascularization encroaches on the hypertrophic chondrocytes, the entire mass is eventually ossified into woven bone (Claes et al., 2012; Bahney et al., 2019). The woven bone formed at the fracture site bridges the two fractured segments and is later remodeled to laminar bone (Hadjidakis and Androulakis 2006; Raggatt and Partridge 2010).

Much is known about how specific signaling cascades, inflammatory markers, the extracellular matrix (ECM) environment, and mechanical stress affect bone repair mechanisms. However, less is known about the role of reactive oxygen species (ROS) in bone repair. At physiologic levels, ROS play important physiological roles in a variety of cell types; however, when ROS levels exceed physiologic levels, they can cause cellular damage and contribute to disease pathogenesis (Kobayashi and Suda 2012; Kalyanaraman 2013; Huang and Li 2020; L.; Zhang et al., 2019; Y.; Zhang et al., 2020; Brown and Griendling 2015). With the more recent discoveries that elevated ROS levels stimulated osteoclast differentiation and influenced osteoblast formation, it is evident that ROS play an important role in regulating the human skeleton via redox signaling pathways (Hyeon et al., 2013, 2; Arakaki et al., 2013).

## 2 Redox Signaling

Free radicals were first described in biology 1954, and since then, the harmful effects of ROS (a broader term encompassing both oxygen molecules with free radicals and non-free radical intermediates) on a wide variety of cellular processes have been well studied (Commoner et al., 1954; Finkel and Holbrook 2000). ROS include superoxide (O_2_^•^), hydrogen peroxide (H_2_O_2_), and hydroxyl radical (^•^OH). More recently, these highly reactive molecules are not just thought to be reapers of havoc, but they are showing to be important molecules regulating downstream signaling cascades. While previously a point of contention among the scientific community, ROS are now widely recognized as second messengers because of their regulated production, the existence of ROS elimination systems, and their target specificity (Raaz et al., 2014). Redox signaling is the general term given to describe the phenomenon of how these molecules mediate signal transduction pathways (Kalyanaraman 2013).

### 2.1 Reactive Oxygen Species Production Machinery

**FIGURE 1:**
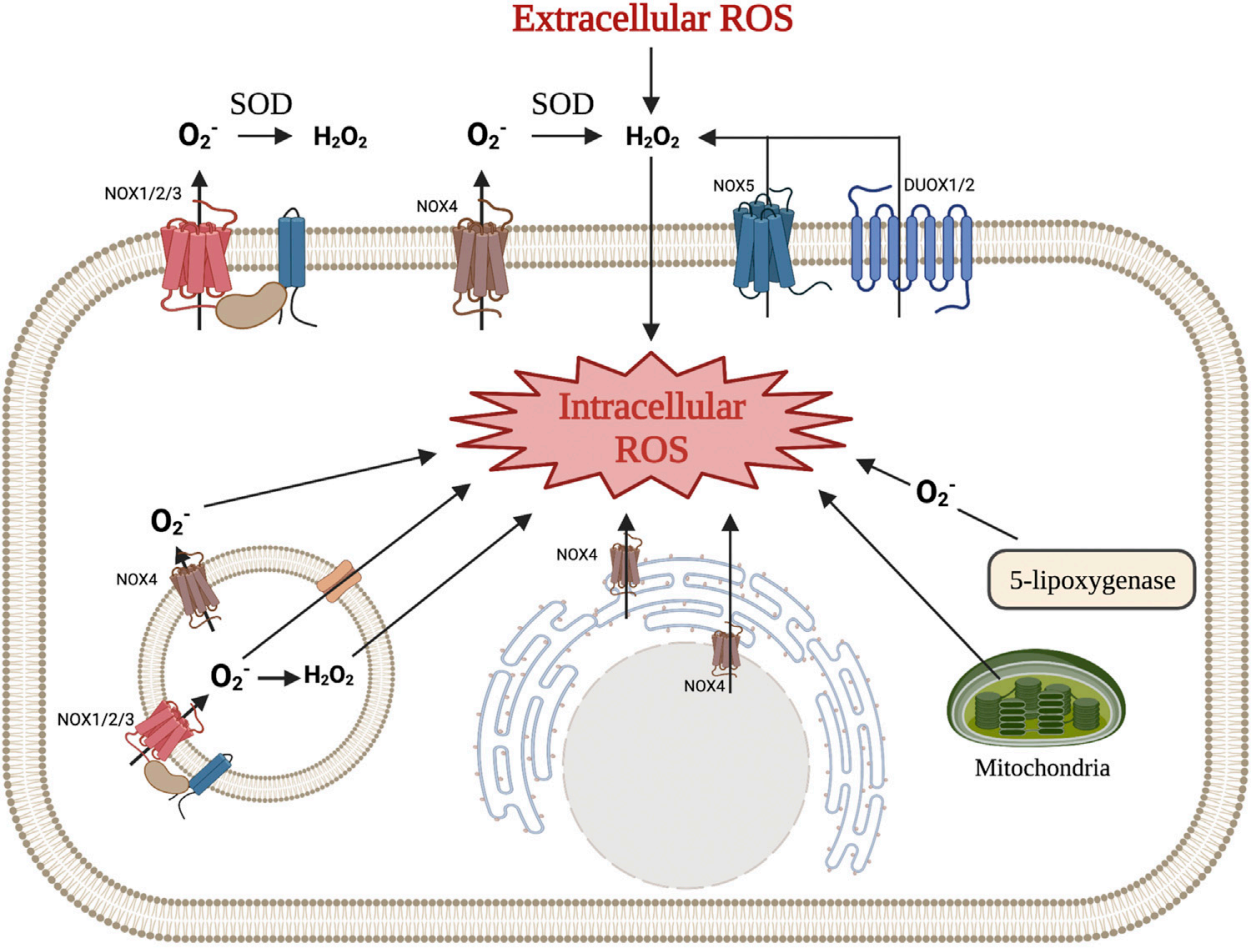
Various mechanisms of intra- and extracellular ROS production. NOX1, NOX2, NOX3, and NOX4 produce superoxide, which is then converted to hydrogen peroxide in the extracellular space. NOX5 and DUOX1/2 produce hydrogen peroxide directly. Hydrogen peroxide produced by these enzymes, along with ROS from other cells, can diffuse across the cellular membrane to function as an intracellular signaling molecule. NOX enzymes can also be found in intracellular membranes (i.e., rough endoplasmic reticulum, endosomes, and nucleus) and produce ROS that can immediately effect signaling pathways. Other mechanisms for intracellular ROS production include the mitochondrial respiratory chain and a byproduct of 5-lipooxygenase. (Illustration created with BioRender.com).

Intracellular ROS are generated by enzymes and exist primarily in the forms of O_2_^•^, H_2_O_2_, and •OH (Lambeth 2004; Lambeth and Neish 2014; Bedard and Krause 2007). Transmembrane NADPH oxidases (NOXs) and the mitochondrial electron transport chain (ETC) are two significant endogenous enzymatic sources of O_2_^•^ and H_2_O_2_ (Parascandolo and Laukkanen 2019; Murphy 2009). These sources, along with others, are illustrated in Figure 1. These molecules have a wide range of downstream targets, and H_2_O_2_ acts as a second messenger that can integrate environmental stimuli, quickly diffuse through membranes, and activate downstream signal transduction cascades (Bedard and Krause 2007; Kobayashi and Suda 2012). Through the dismutation reaction mediated by either potassium iodide or superoxide dismutase (SOD), O_2_^•^ can be reduced to H_2_O_2_, which can be further reduced to oxygen and water (Dröge 2002; Panday et al., 2015). Enzymes such as glutathione peroxidase (Gpx), catalase, and peroxide redox proteins (PRX), as well as other antioxidants (i.e. Vitamin E), are important ROS scavengers that ultimately convert ROS to oxygen and water to help maintain redox homeostasis (L. Zhang et al., 2019; Y. Zhang et al., 2020; Brown and Griendling 2015; Hu et al., 2018). ROS-induced protein modifications can also be reversed by specific antioxidant defense proteins, including glutaredoxins, thioredoxins, and thioredoxin reductases, making them important in the prevention of ROS-induced cellular damage (Drazic and Winter 2014).

In bone tissue, redox signaling has an important role in bone remodeling and bone repair (Garrett et al., 1990; Lean et al., 2003; X.; Sun et al., 2020, 2; Deng et al., 2019), and research has shown that oxidative stress, which leads to aging and estrogen deficiency, may be one of the most critical factors contributing to bone loss (Almeida et al., 2007b; Manolagas and Almeida 2007). Of more focus in this review, redox signaling is also triggered following bone fracture to regulate bone healing and regeneration by targeting resident stromal cells, osteoblasts, osteoclasts and endothelial cells (Ito and Suda 2014; K.-M.; Kim et al., 2019; Kang and Kim 2016; Yamamoto et al., 2020).

## 3 Redox Signaling and Fracture Healing

### 3.1 Role of Reactive Oxygen Species in the Inflammatory Phase and Neovascularization

**FIGURE 2:**
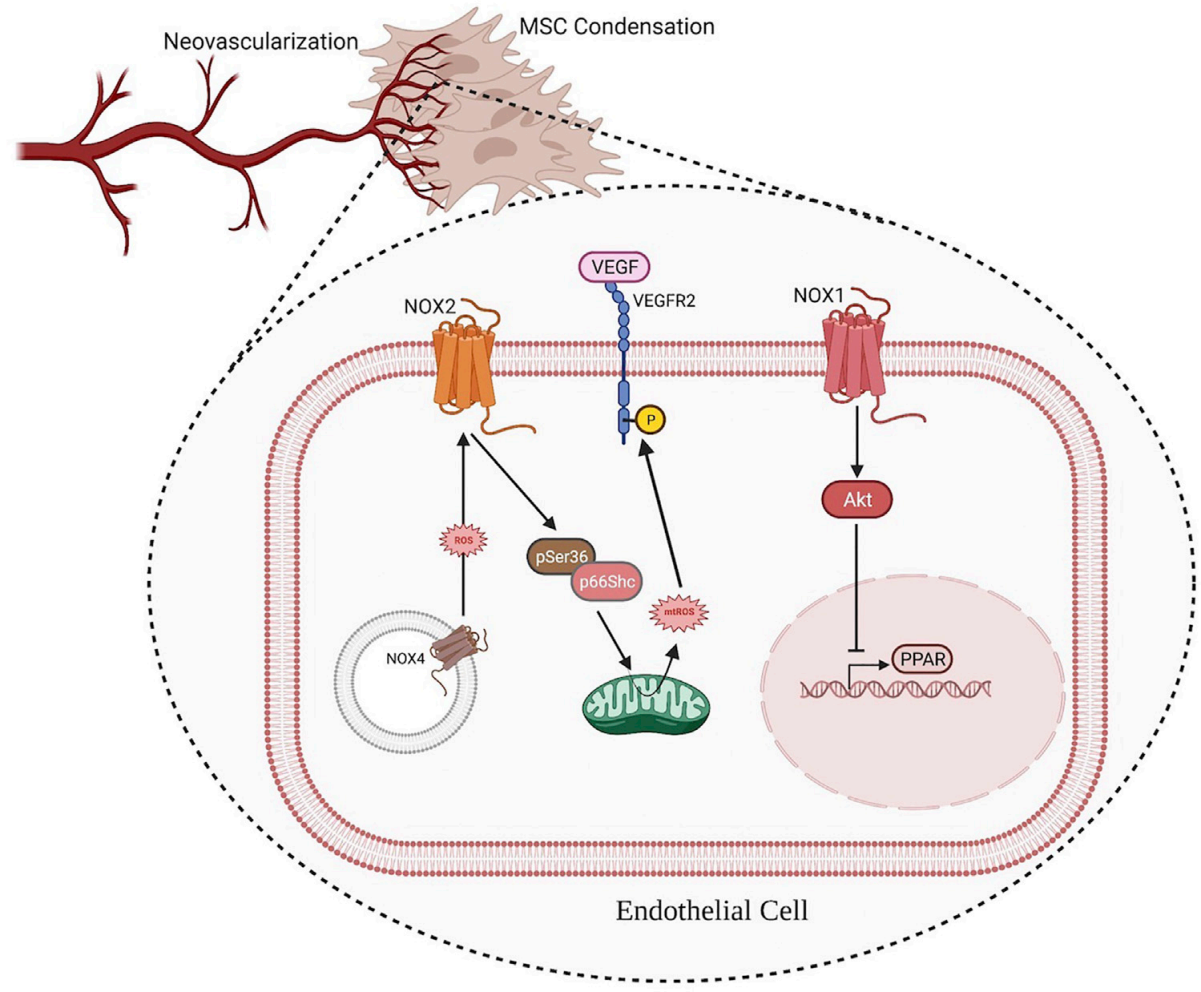
ROS produced by NOX1 contributes to the activation of the Akt pathway and downregulation of the anti-angiogenic nuclear receptor PPAR, resulting in an event associated with capillary tube formation. Additionally, intracellular membrane-associated NOX4 produces ROS, which activates NOX2 and the down-steam factor pSer36-p66Shc to promote mtROS production. The ROS produced by mitochondria further enhances VEGF signaling, thus improving angiogenesis. (Illustration created with BioRender.com).

From distraction osteogenesis and other models fracture healing, the process of osteoblast differentiation and bone mineralization is closely coupled to neovascularization (Percival and Richtsmeier 2013). In fact, the invasion of new blood vessels into the mesenchymal condensations is necessary for the mineralization process to begin (Percival and Richtsmeier 2013; Tomlinson et al., 2013). The decision whether to pursue intramembranous ossification–rather than endochondral ossification–seems to be dependent on closeness of the nearest vascular supply (Percival and Richtsmeier 2013). In particular, NOX1 contributes to the Akt pathway’s activation and downregulation of the anti-angiogenic nuclear receptor peroxisome proliferator activated receptor (PPAR), resulting in an event associated with capillary tube formation (Manea 2010; Bir et al., 2013; Huang and Li 2020). On the other hand, NOX2 plays a slightly different role in angiogenesis; it has been shown to promote endothelial cell migration, mobilize endothelial progenitor cells, and exert pro-angiogenic functions in response to vascular endothelial growth factor (VEGF) (Thakur et al., 2010; Schröder et al., 2012). The proliferation and migration of endothelial cells (ECs) could be enhanced by the Upregulation of the Nox4 expression, which results in the activation of receptor tyrosine kinases and the Erk pathway. Kim et al. found a novel positive feed-forward ROS-induced ROS release mechanism in which H_2_O_2_ (derived from NOX4) partially activates NOX2, thereby promoting mitochondrial ROS (mtROS) production through pSer36-p66Shc, which further enhances the ROS-dependent VEGFR2 signaling pathway in ECs. Through this mechanism, the Nox4/Nox2/pSer36-p66Shc/mtROS axis drives an angiogenic switch (Y.-M. Kim et al., 2017) (Figure 2).

The role of mitochondria in redox signaling and VEGF signaling has been recently more elucidated. UQCRB (a subunit of complex III in the mitochondrial respiratory chain) can positively regulate VEGFR2 signaling by increasing levels of mtROS as a key regulator of VEGF signaling in ECs. By the above approach, UQCRB also regulates the migration of ECs *in vitro* (Jung and Kwon 2013).

### 3.2 Effect of Reactive Oxygen Species on Mesenchymal Stem Cells Function

While damaged blood vessels and the hypoxic environment itself kickstarts angiogenesis, MSCs play a large part as well. For intramembranous ossification, it is important that the condensation of MSCs signal in a timely manner to the vascular cells so that osteogenic differentiation and mineralization can occur. MSCs can directly signal to assist angiogenesis in fracture healing by interaction with a variety of factors.

#### 3.2.1 Vascular Endothelial Growth Factor

**FIGURE 3:**
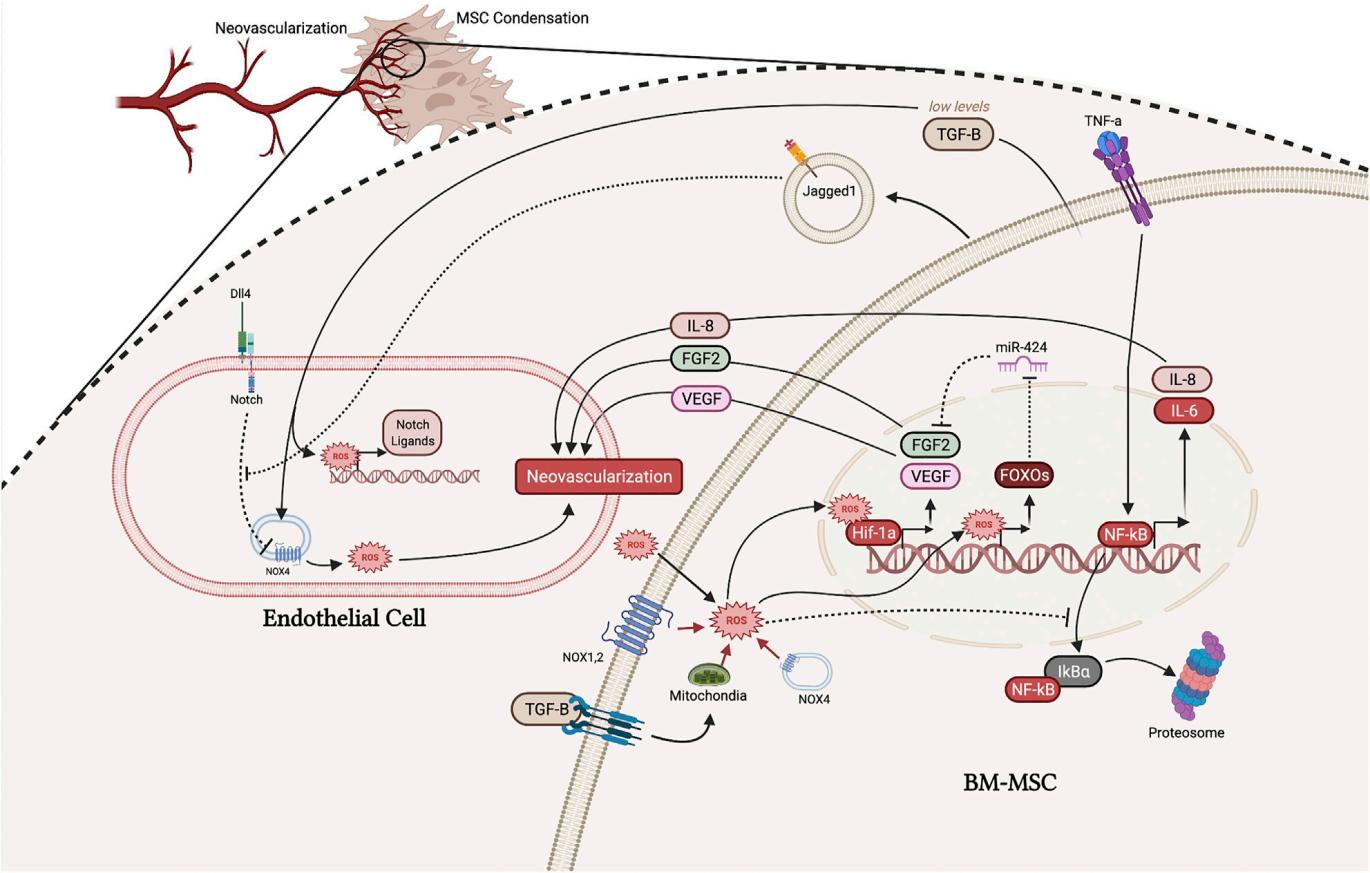
Interaction between ECs and MSCs and in the early microfracture environment. ROS from a variety of sources, including the mitochondria, NOX enzymes, TGF-β signaling, and the extracellular environment, can stabilize Hif-1α resulting in upregulation of FGF-2 and VEGF, which promote neovascularization. ROS also directly upregulate FOXOs which inhibits miR-424, further promoting the functioning of FGF-2. Increased intracellular ROS promotes NF-κB signaling, thus upregulating IL-8 which promotes neovascularization but potentially inhibits intramembranous ossification. Jagged1 and TGF-β secreted from MSCs also function to promote neovascularization by promoting NOX4-generated ROS. (Illustration created with BioRender.com).

As previously mentioned, angiogenesis and intramembranous bone formation are closely coupled, such that angiogenesis proceeds osteogenesis (Ko and Sumner 2021). VEGF is the primary signaling molecule allowing osteoprogenitors to both signal to ECs and initiate the release of growth factors needed for the differentiation process (Ko and Sumner 2021). In a mouse model, when VEGF activity was blocked in osterix-positive cells, postnatal intramembranous ossification was significantly impaired (Ko and Sumner 2021; Buettmann et al., 2019). ROS play an important role in regulating VEGF secretion in MSCs. When preconditioned to hypoxic environments, MSCs enhance their angiogenic effect by upregulating VEGF (Black et al., 2008). Increased levels of ROS stabilize hypoxia-inducible factor 1 α (Hif-1 α ), preventing its degradation, and allowing it to transcribe a host of hypoxia-related proteins, including VEGF (Figure 3) (Black et al., 2008). This ROS/Hif-1α/VEGF signaling pathway is observed in cells involved in osteogenesis, and the disruption of ROS can prevent normal VEGF signaling (F.-S. Wang et al., 2004; V. T. Nguyen et al., 2020). For example, when ROS-scavenging enzymes are inhibited, osteoblasts drastically reduce their transcription of VEGF, thus impairing angiogenesis during bone repair (F.-S. Wang et al., 2004).

#### 3.2.2 Fibroblast Growth Factors

Fibroblast growth factors (FGFs) are a large family of signaling factors that function in all stages of fracture healing and have been shown to be important in MSC differentiation, skeletal vascularization, and osteoblast recruitment (Schmid et al., 2009). FGF2 is the principle FGF expressed in distraction osteogenesis and is known to enhance EC survival, migration, and proliferation (Schröder 2019; Pacicca et al., 2003; Schmid et al., 2009). After FGFs bind the FGF receptor on ECs, phosphoinositide 3-kinase (PI3K) acts to increase ROS via NOX2. ROS produced from the FGF-NOX2 pathway has then been shown to inactivate phosphatases, allowing pro-angiogenic signaling pathways to be active longer (Schröder 2019). In vascular smooth muscle cells, FGF-2 has been shown to stimulate its own expression via a ROS-dependent mechanism (Black et al., 2008). ROS produced through the FGF-PI3K-ROS pathway functions to stabilize and increase expression of Hif-1α, which directly increased the activity at the FGF-2 promoter (Figure 3) (Black et al., 2008). Therefore, FGF-2 uses ROS as a key signaling molecule to upregulate its own expression.

FGF-2 secreted from MSCs, as well as other cell types, functions to stimulate angiogenesis in ECs and vascular smooth muscle through a ROS-dependent process. Given that the FGF receptor 1 and 2 are known to signal through the PI3K-AktMDM2 pathway, it is likely that FGF-2 can also stimulate its own expression in MSCs, though no studies have been done to show this possibility (Coutu et al., 2011). Li et al. showed that MSCs in fact have ROS sensing mechanisms to regulate the expression of FGF-2 by suppressing the microRNA miR-424 (L. Li et al., 2017). miR-424 has been shown to negatively regulate bone formation under oxidative stress by inhibiting FGF-2. However, this group found that MSCs upregulate forkhead box O 1 transcription factor (FOXO1) in response to ROS, and FOXO1 then inhibits the production of miR-424 (Figure 3) (L. Li et al., 2017; Siqueira et al., 2011, 1). In sum, MSCs can sense ROS levels and subsequently enhance the release of FGF-2; both ECs and MSCs can sense their oxidative environment and upregulate FGF-2 to improve neovascularization of the microfracture environment.

As discussed, low levels of ROS can enhance the early neovascularization of the fracture site; however, high levels of ROS in MSCs can be detrimental and induce rapid senescence (Ludin et al., 2014). To combat the high oxidative stress of the fracture microenvironment, MSCs possess numerous mechanisms to reduce and manage ROS levels, including high levels of antioxidants, glutathione, and other ROS scavengers (Ludin et al., 2014). One study found that FGF-2 may promote low levels of ROS and maintains stemness of MSCs (Ludin et al., 2014). In a mouse model, FGF-2 *reduced* ROS levels in a PI3K-Akt-MDM2 dependent manner, thus increasing proliferation and self-renewal of MSCs (Coutu et al., 2011). Therefore, low levels of FGF-2 and abundant ROS scavenging mechanisms help MSCs maintain optimum levels of ROS to promote proper MSCs function. Interestingly, FGF plays a different role in the microfracture environment, and both ECs and MSCs can use ROS to upregulate FGF-2 to favor the neovascularization of the fracture site. MSCs seem to favor the expression of FGF-2 in response to ROS, however they may not use an autoregulatory positive feedback loop like ECs. Instead, FGF-2 may in fact still use PI3K, but to reduce levels of ROS so that they can function properly. More rigorous *in vitro* and *in vivo* studies need to be done to further investigate the role of FGF-2 and ROS in the oxidative microfracture environment.

#### 3.2.3 Notch

Notch signaling is one of the most representative pathway in regulating tip cell specification (Suchting and Eichmann 2009). Recent study showing the increase in notch ligands are dependent on Hif-1α, a upstream of Notch pathways (Ciria and Nahuel, 2017). Hif-1α is activated by elevated ROS levels leading to the increase of Notch pathway and subsequent angiogenesis. However, given that there are 4 known Notch receptors that can be activated by 5 different Notch ligands, its role is extremely complex (Ciria and Nahuel, 2017). For example, Notch Ligand Delta-like 4 (Dll4) and Jagged1 have distinct spatial expression patterns and opposing functional roles in regulating angiogenesis (Benedito et al., 2009). Dll4, which is activated by VEGF in angiogenesis, contributes to *inhibiting* the sprouting of endothelial tip cells (Benedito et al., 2009). On the contrary, Jagged1 antagonizes the Dll4-Notch signaling, which promotes angiogenesis (Benedito et al., 2009; Hai et al., 2018).

Furthermore, it has been shown that ROS regulate the notch pathway’s role in angiogenesis (Cai et al., 2014; Gonzalez-King et al., 2017). Studies have shown that Notch signaling mediates ROS production in Nox4-dependent manner. In ECs, blocking notch signaling could upregulate the expression of Nox4 and increase ROS generation while using ROS scavengers could result in abolished Notch blockade-induced EC proliferation, migration, and adhesion (Cai et al., 2014). Therefore, the stabilization of Hif-1α in MSCs may result in a decrease in EC’s intracellular ROS, which would inhibit angiogenesis. In contrast, Jagged1 is a notch ligand that may *enhance* angiogenesis in the hypoxic fracture microenvironment. Gonzales-King et al. made a step towards confirming this notion by showing that MSCs can package and secrete Jagged1 via exomes, which had a pro-angiogenic effect *in vitro* and *in vivo* (Figure 3) (Gonzalez-King et al., 2017). However, the mechanism by which notch signaling influences angiogenesis is still unclear since some notch ligands have opposing actions.

#### 3.2.4 TGF-β

Transforming growth factor beta (TGF-β) is an important cytokine in the early initiation of fracture healing. However, its role in neovascularization of the fracture cite is complex, with some studies showing low levels of TGF-β secreted by MSCs stimulates endothelial tube formation while others show the opposite (Kasper et al., 2007; Myoken et al., 1990). From more recent studies, the role of TGF-β in neovascularization of the fracture site may be elucidated by its effects on ROS in ECs and MSCs. For ECs, TGF-β has a positive effect on NOX4; ROS produced by NOX4 then go on to directly stimulate angiogenesis and facilitate the upregulation of Notch ligands, Dll4, Notch1, and Jagged1 (Figure 3) (Yan et al., 2014, 4). It is also possible that Jagged1 functions to antagonize the Dll4-Notch pathway, resulting in a positive feedback loop to further stimulate NOX4 (Ciria and Nahuel, 2017; Gonzalez-King et al., 2017). These other notch ligands produced by ECs can then act directly to promote the osteogenic differentiation of MSCs to heal the fracture (Dishowitz et al., 2012; Ciria and Nahuel, 2017; Gonzalez-King et al., 2017). Thus, TGF-β seems to have an overall favorable effect on angiogenesis and early fracture repair.

However, at higher levels, one study found that TGF-β1 has a negative effect on MSCs by upregulating mitochondrial production of ROS and increasing senescence (J. Wu et al., 2014). While this study only investigated the *in vitro* effect of TGF-β on MSCs, these findings may provide some insight to TGF-β’s role in the initial fracture healing response. At lower levels, TGF-β -induced production of ROS may facilitate the pro-angiogenic capacity of MSCs by many of the mechanisms mentioned throughout this section (Figure 3). However, at higher levels of TGF-β, the overproduction of ROS may switch MSCs toward an apoptotic phenotype (J. Wu et al., 2014). From *in vivo* studies, the levels of TGF-β gradually increases throughout the fracture healing process over the first 14 days (Poniatowski et al., 2015). While this topic is lacking rigorous experiments, it is reasonable to speculate that low levels of TGF-β functions to facilitate proangiogenic levels of ROS, while higher levels of TGF-β are more favorable in later stages of fracture healing, where an increase in apoptosis may be beneficial in producing calcium deposits for bone nodule formation.

#### 3.2.5 NF-κB and IL-8

One of the best described mechanisms by which MSCs initiate angiogenesis is through the upregulation and secretion of interleukin 8 (IL-8) by inflammatory factors such as tumor necrosis factor a (TNF-α) (J. Wang et al., 2015; Kwon et al., 2013). IL-8 is under the influence of the nuclear factor-kappa B (NF-κB) pathway, and NF-κB has been shown to be greatly affected by ROS levels. Increased ROS levels promotes the proteosome degradation of IκBa, which normally functions to inhibit NF-κB from upregulating inflammatory cytokines (G. Li et al., 2019; Bai et al., 2020). Thus, by promoting the degradation of IκBα, ROS can promote the upregulation of IL-8 (Figure 3).

While IL-8 functions to initiate angiogenesis, it also is a potent chemoattractant and recruits osteoprogenitor cells to the fracture site (Lin et al., 2019). By both initiating angiogenesis and recruiting MSCs, IL-8 would seem to play an important role in intramembranous ossification. However, this may not be the case. Recent literature shows that IL-8 induces chondrogenesis and has little ability to promote osteogenesis and mineralization (Lin et al., 2019). While Yang et al. found that IL-8 increased osteogenesis in a large bone defect mouse model after 14 days, another recent study found that this effect may be due to IL-8’s ability to rapidly induce chondrogenesis and synergize with bone morphogenic protein (BMP) 2 signaling (A. Yang et al., 2018; Lin et al., 2019). Therefore, IL-8 potentially favors endochondral ossification, seen in larger fracture site, and likely disrupts intramembranous ossification.

As mentioned above, ROS can favor endochondral ossification via the expression of inflammatory cytokines such as IL-8. This effect is well explained by the size of bone defect; for example, the greater oxidative stress seen in larger defects allows intracellular ROS levels to exceed ROS scavenging mechanisms. This increased intracellular ROS then upregulate IL-8 to promote chondrogenesis. Therefore, the decision to pursue intramembranous versus endochondral ossification is greatly dependent on the MSC’s ability to regulate and sense ROS levels. More studies need to investigate the levels of ROS at which IL-8 is upregulated. Further, the existing literature utilizes cell culture and large-sized bone defects to investigate the effects of IL-8 on bone healing (A. Yang et al., 2018, 8; Lin et al., 2019), so there is a need for new bone defect models to assess the role ROS and IL-8 in intramembranous ossification.

### 3.3 Bone Morphogenic Protein Signaling

Vascular cells are also known to upregulate BMPs in response to hypoxia and VEGF, functioning as a sensor of the fracture microenvironment and delivering the needed factors for MSCs to differentiate (Percival and Richtsmeier 2013). BMP2 is necessary for MSCs to initiate fracture repair, thus its release from vascular cells during hypoxia is a vital signal to kickstart intramembranous ossification. Studies show that silencing BMP2 expression results in fractures that do not heal in a mouse model, as a direct result of MSCs not differentiating to osteoblasts (Tsuji et al., 2006).

There is also an important interplay between ROS and BMP signaling in pre-osteoblasts. BMPs have been shown to upregulate NOX enzymes, thus increasing intracellular ROS in MSCs (Mandal et al., 2011; Sánchez-de-Diego et al., 2019). These ROS can then function to upregulate BMPs, notably BMP2 and BMP4 in MSCs, by increasing the activity of NF-κB signaling (Csiszar et al., 2005). This expression of BMPs by MSCs seems to be of most importance in fracture healing since fracture healing was severely impaired by knocking out BMP2 gene expression in an animal model, whereas knocking out BMP2 expression in osteoblasts and chondrocytes only delayed fracture healing (McBride-Gagyi et al., 2015, 2; Muinos-López et al., 2016). Therefore, NOX dysfunction may have deleterious effects on MSC’s ability to differentiate by downregulating BMP2 expression.

ROS and BMPs both play an important role in enhancing osteogenic differentiation of MSCs. Muinos-López et al. also showed that hypoxia and the regulation of ROS play a large role in BMP2 production by MSCs (Muinos-López et al., 2016). In their experiment, they found that hypoxia alone was insufficient to induce BMP2 expression in human periosteal-derived MSCs, and hypoxia with inflammatory cytokines greatly increased BMP2 activity, but not greater than just inflammatory cytokines alone. However, when the ROS-scavenger inhibitor PX-12 was added to the hypoxic and cytokines group the levels of BMP2 significantly decreased (Muinos-López et al., 2016). From their work, it seems that ROS are necessary for the inflammatory cytokines to adequately upregulate BMP2 signaling. Further, they also tested this in a fracture healing mouse model and found that PX-12 significantly reduced Bmp2 signaling, resulting in impaired fracture healing and an atrophic-like nonunion (Muinos-López et al., 2016).

However, given that BMPs signals to activate NOX which then further upregulate BMPs, it is easy to see how this positive regulatory loop can get out of hand, leading to high oxidative stress. It has been extensively published that high levels of ROS are damaging to osteoprogenitors, resulting in decreased proliferation, increased apoptosis, and decreased osteogenic differentiation (X. Bai et al., 2004; Benameur et al., 2015; Denu and Hematti 2016). The above findings suggest there is a delicate balance between MSC intracellular ROS and ROS scavenger mechanisms to allow for optimal ROS-induced BMP2 signaling, while also suppressing ROS to manageable levels.

### 3.4 FOXO and Wnt Signaling

Activation of the Wnt/ β -catenin pathway is generally recognized as enhancing the osteogenic and chondrogenic potential of MSCs, thus important during the fracture healing process (Atashi et al., 2015). In fact, studies have shown this pathway is upregulated during the entire fracture process (H. Xu et al., 2014; Chen et al., 2007). To describe briefly, after an extracellular Wnt ligand binds to the frizzled seven pass transmembrane receptor, the degradation of β -catenin is inhibited, allowing its migration to the nucleus to bind T-cell factor/lymphoid-enhancing factor (Tcf/Lef) to transcribe Wnt effector genes known to upregulate osteogenesis (H. Xu et al., 2014). ROS have been shown to interfere with this signaling cascade, hence impairing the osteogenesis of MSCs. Almeida et al. discovered that the decrease in Wnt effector genes, such as *osteoprotegerin* and *Axin2* is associated with the increase in ROS seen in aged mice (Almeida et al., 2007a).

The above finding then led to the discovery that increased ROS upregulates the transcription of the FOXO family of transcription factors. FOXOs, including FOXO1, FOXO3a, FOXO4, and FOXO6, require β -catenin for the transcription of their target genes (Atashi et al., 2015). In other words, FOXOs steals β -catenin from the Wnt/ β -catenin pathway and uses it to increase transcription of a variety of gene, of which include antioxidant and ROS-scavenging enzymes. In one study, osteoblast number and bone mass was increased in mice deficient in FOXOs (Iyer et al., 2013). Furthermore, mice treated with H_2_O_2_ was shown to increase FOXO association with β -catenin (Almeida et al., 2007a). On the contrary, Ambrogini et al. showed that mice deficient in FOXOs had elevated oxidative stress and high levels of osteoblast apoptosis, whereas mice with FOXO overexpression had less osteoblast apoptosis and significantly increased bone formation and vertebral bone mass (Ambrogini et al., 2010).

From the studies above, both Wnt and FOXO signaling is important for fracture healing. However, when ROS levels are elevated to pathologic levels, these two signaling pathways interfere with each other. At physiologic levels of ROS, the constitutive level of ROS-scavengers and antioxidants (downstream of FOXO pathway) are sufficient to prevent cellular damage. However, when ROS are elevated, the FOXO pathway is stimulated to provide more ROS-scavenging power. While this is good for cellular health, it inhibits the osteogenic potential of osteoprogenitors through inhibiting the Wnt/ β -catenin pathway.

Up to this point, it is evident that ROS are necessary for fracture healing to take place. However, their levels must be tightly controlled, and increased levels can inhibit or delay the fracture healing process.

## 4 Harnessing Reactive Oxygen Species for Bone Regeneration

As discussed extensively in the first half of this review, ROS plays an important role in many pathways involved in the early phase of fracture repair. However, it is also clear that pathologically elevated ROS levels contribute to poor skeletal healing, such as in diabetes. Diabetes claims one of the top spots of diseases affecting Americans, and it is one of the most well researched pathologies related to excess ROS, which has been clearly linked to poor outcomes. Not only does the increased oxidative stress add to the vast tissue damage and complications, but the overproduction of ROS also limits therapeutic potential by negatively affecting stem cell production. More recently, the direct effects of ROS in inducing microangiopathy in bone marrow has been highlighted as a mechanism of diabetes in both contributing to the decline of the disease and preventing healing (Mangialardi et al., 2014). Additionally, the oxidative stress environment of diabetes, or any other pathology, contributes to osteoblast and osteoclast dysfunction, resulting in reduced bone mass and impaired fracture healing (Jiao et al., 2015). Bone specific diseases, such as osteoporosis and bone tumors, along with joint inflammatory diseases, including rheumatoid arthritis and ankylosing spondylitis, have also been linked to an increase in ROS (Wauquier et al., 2009).

Many of the pathologies discussed are associated with an overproduction of ROS, which suggest that certain levels above a threshold may exacerbate the disease state or prevent healing. It is acknowledged that some physiological level of ROS must exist for initiation of distinct cell processes, pointing to some minimum amount that may also need to be present for favorable outcomes. Understanding how ROS levels affect pathological mechanisms and healing allows for better optimization of microenvironment conditions and the development of therapies. We discuss below how therapies may be used to either increase or decrease ROS to improve fracture healing.

### 4.1 Utilizing Exogenous Antioxidants to Improve Fracture Healing

Antioxidants and ROS-scavenging enzymes seem to hold obvious therapeutic promise as targets to decrease ROS and improve fracture healing. However, as previously discussed, ROS are necessary for the bone healing process to occur, while the levels of ROS may determine the bone healing type and quality.

#### 4.1.1 Vitamin C

Vitamin C is known to play a critical in musculoskeletal healing and serves as a cofactor for prolyl hydroxylase and lysyl hydroxylase, two enzymes necessary for proper collagen three-dimensional conformation. Further, Vitamin C is a powerful antioxidant and a stimulator of osteoblast growth and differentiation (Lee et al., 2017). Therefore, it is reasonable to suspect that supplementation with Vitamin C would improve fracture healing. However, clinical evidence does not fully support this statement. In a large systemic review, DePhillipo et al. reported conflicting clinical evidence for use of Vitamin C in enhancing fracture healing (DePhillipo et al., 2018). One study found that patients who were treated with vitamin C after open reduction internal fixation had higher plasma levels of alkaline phosphatase (ALP) and Osteocalcin, suggesting higher BMD and faster healing with antioxidant supplementation (Sandukji et al., 2011). On the other hand, another group found that vitamin C had no significant effect on time to fracture-healing at day 50 post-surgery of distal radial fractures (Ekrol et al., 2014). Given that the size of fracture, injury mode, and fracture management modality all contribute to the level of ROS at the fracture site, it is difficult to tease out the effect of vitamin C in the latter study. For example, vitamin C may have greater effect on fracture healing after an open reduction internal fixation, compared to an external fixation, since the levels of ROS may be drastically different post-surgery. Better controls need to be in place for clinical studies to better understand the effects of vitamin C on fracture healing.

Similar to the previous clinical studies, animal studies also showed conflicting data on the improvement of fracture healing with vitamin C (DePhillipo et al., 2018). Giordano et al. found that vitamin C supplementation had no effect on histological features of fracture healing at week 2, 4, and 6 (Giordano et al., 2012). However, Sarisozen et al. analyzed fracture repair at week 2 and 3 and saw accelerated fracture repair (Sarisözen et al., 2002). Similarly, Yilmaz et al. measured fracture repair at even earlier time points (5, 10, 15, and 20 days post-fracture) and found vitamin C accelerated fracture healing, while having no effect on end quality of fracture healing (Yilmaz et al., 2001). Taken together, vitamin C may play an important role in accelerating bone healing during the inflammatory phase of fracture healing, when ROS levels are the highest. Interestingly, a recent metanalysis by Sun et al. found that increasing dietary vitamin C by 50 mg/day would decrease risk of hip fracture by 5% (Y. Sun et al., 2018). Given that aging is associated with increased levels of ROS, is it reasonable to hypothesize that vitamin C may reduce intracellular ROS, allowing aged osteoprogenitors to better handle the higher levels of oxidative stress seen in aging. Therefore, vitamin C may be a promising supplement, not only in fracture healing, but also in the prevention of age-associated bone loss and fracture.

#### 4.1.2 *α*-tocopherol

*α*-tocopherol (AT) is a vitamin E isomer that has antioxidant properties and effects on various metabolic systems. AT’s effect on bone healing is debated, as some studies show that it does not improve fracture repair while others show the opposite (Sarisözen et al., 2002; Durak et al., 2003; Mohamad et al., 2012). More recent insight compares AT to a double-edged sword. At high doses, AT may have prooxidant effects, block entry of other vitamin E isoforms, and interfere with vitamin K metabolism (Chin and Ima-Nirwana 2014). At lower doses however, AT has antiosteoporotic effects and scavenges ROS (Shuid et al., 2011; Chin and Ima-Nirwana 2014). While AT’s effect on bone health is relatively unknown, one finding among the literature seems to be consistent: AT is protective of bone and enhances bone healing under stressful conditions.

Smith et al. studied the effect of AT on hindlimb-unloading and found that high doses (500 IU/kg) produced lower trabecular number and decreased bone volume in mice compared to the lower doses (15 IU/kg and 75 IU/kg) (Smith et al., 2005). However, the higher dose of AT prevented the hindlimb-unloading bone loss been in osteoporotic mice (Smith et al., 2005). In another study, high doses of AT prevented bone loss and improved bone structure of aged mice (24 month-old), while it had not effect on bone structure of younger mice (6 month-old) (Arjmandi et al., 2002). The authors also showed AT increased the mRNA expression of insulin-like growth factor 1 (IGF-1), osteocalcin, and type-1 collagen in the bones of both young and old mice (Arjmandi et al., 2002). Additionally, Zhang et al. noted that smokers had a lower prevalence of fractures if they had higher dietary intake of AT (J. Zhang et al., 2006). By increasing bone mass in an osteoporosis model, improving bone health of aged mice, and preventing fractures in smokers, AT seems to provide a profound protective effect in environments of high oxidative stress.

Unlike most lipid-soluble vitamins, AT is inserted in the lipid cell membrane of cells and lipoproteins, as well as acts ubiquitously throughout the body at high concentrations (Miyazawa et al., 2019). Therefore, at higher concentrations of AT, resulting in higher plasma levels, it is possible that AT could be inhibiting the physiological levels of ROS that are required for bone healing (Baxter et al., 2012). Whereas, in environments of constitutively high ROS, this increase in AT provides a net sum benefit when extracellular ROS levels are harmful to the healing process.

To fit with the hypothesis that the most important aspect of fracture healing is to lower intracellular ROS to maintain optimal function of regulation of osteoprogenitors, AT should decrease ROS in osteoprogenitors and MSCs. A more recent study by Bhatti et al. confirmed that AT decreases oxidative stress in both adipose and bone marrow-derived MSCs, supporting the positive effect of AT on fracture healing (F. U. R. Bhatti et al., 2017; F. U. Bhatti et al., 2017). Additionally, AT has been shown to suppress cyclooxygenase activity by preventing the hydrolysis (activation) of phospholipase A_2_ (Miyazawa et al., 2019). This inhibition of phospholipase A_2_ may aid in the decrease of intracellular ROS of osteoprogenitors and contribute to the protective effects of AT as shown by Smith et al. (2005). All in all, the direct ability of AT to improve fracture healing is complicated and is potentially dependent on dose and level of bone injury. Durak et al. found that AT improves the later stages of bone healing in a rabbit model of fracture healing, and a more recent paper found that AT can improve the osteointegration of stainless steel metal implants by reducing postoperative stress in a rat model (Durak et al., 2003; Savvidis et al., 2020). Therefore, AT holds significant promise in enhancing fracture healing. From the present evidence, its seems that AT is beneficial in suppressing ROS in environments of high oxidative stress, which allows normal bone healing. However, the literature suggests AT may be detrimental to normal fracture repair, likely by suppressing the healthy, physiologic levels of ROS. More well-controlled studies need to be performed to look at AT’s direct role in normal fracture repair to see if beneficial effects can be seen at lower doses of AT.

#### 4.1.3 N-acetyl Cysteine

N-acetyl cysteine (NAC) is a small molecular weight, amino acid derivative antioxidant that can be rapidly transported into the cytoplasm (Yamada et al., 2013). These qualities allow NAC to aid in the scavenging of ROS inside the intracellular space. Further, it has been shown that NAC is catabolized in the cytoplasm and leads to the generation of sulfane sulfur species, which likely perform the bulk of the antioxidant and cytoprotective functions after NAC treatment (Ezeriņa et al., 2018). It is unlikely that NAC by itself significantly contributes to ROS-scavenging, given that the NAC reaction has a low rate constant (Benrahmoune et al., 2000; Ezeriņa et al., 2018).

Further, Yamata et al. showed that NAC directly enhances ALP activity, collagen deposition, and other bone-related markers in osteoblastic cells in culture (Yamada et al., 2013). This group also found that by infusing a collagen sponge with NAC could drastically improve bone healing when implanted into a critical-sized cortical bone defect (Yamada et al., 2013). The osteogenesis enhancing effect of NAC is clear; however, it is not clear whether this effect is due to direct enhancement of osteoblastic differentiation or by decreasing the constitutive level of ROS, allowing optimal MSC function and differentiation. Roper et al. used alcohol to delay fracture healing in mice. This group has shown alcohol induced oxidative stress upregulates FOXO production, which antagonizes Wnt signaling to impair fracture healing (Roper et al., 2016). By administering NAC, they found that ROS levels were suppressed to levels that allowed FOXO levels to decrease, restoring quality of endochondral ossification (Roper et al., 2016). Duryee et al. had similar findings, showing that NAC decreased ROS in alcohol-fed mice and restored the normal innate immune response to fracture healing (Duryee et al., 2018). Taken together, NAC has profound antioxidant actions intracellularly and relatively low actions extracellularly, which make it a promising molecule to suppress ROS levels in osteoprogenitors while not significantly effecting the physiologic levels of ROS in the fracture environment. Further studies need to be conducted that more closely investigate whether NAC is acting primarily to suppress intracellular ROS of osteoprogenitors and immune cells, or if is acting to suppress ROS in the extracellular space of the fracture environment.

### 4.2 Modulating Intracellular Antioxidants to Improve Fracture Healing

Using antioxidants such as vitamin C, AT, and NAC hold significant promise for promoting healthy fracture and bone healing; however, these broad antioxidants have the potential to interrupt physiologic levels of ROS that are necessary for the healing process. A likely better approach to improving fracture healing and bone health is to target intracellular mechanisms of ROS-scavenging. Nuclear factor erythroid 2-related factor-2 (Nrf2) is a key factor that positively regulates the expression of antioxidants and ROS-scavenging enzymes through binding antioxidant response element (ARE) (Y. Kubo et al., 2019, 2). Nrf2 signaling plays an essential role in the fracture healing response, as Lippross et al. has shown that knocking out Nrf2 in mice significantly retards callus formation (Lippross et al., 2014).

It has been shown that Nrf2 is locally upregulated in the fracture environment and VEGF stimulates Nrf2 activity, suggesting that Nrf2 is a protective mechanism of osteoprogenitors to control intracellular ROS levels during this stressful process (Kweider et al., 2011). However, the exact role of Nrf2 in fracture repair is debated. Several studies have found that overactivation of Nrf2 is detrimental to bone healing by inhibiting osteoblast differentiation (Hinoi et al., 2006; Kanzaki et al., 2013). So, much like the antioxidants discusses previously, too much of a good thing is not necessarily good, and it is likely the regulation of Nrf2 during various stages of fracture healing that is of primary importance. Supporting this statement, the upregulation of Nrf2 signaling has been shown to be more beneficial in models of excessive oxidative stress, such as in models of osteoporosis, heavy alcohol intake, type-2 diabetes, and smoking (H. Li et al., 2018; Y. Kubo et al., 2019; Aspera-Werz et al., 2018; Ibáñez et al., 2014, 2). In a more recent study, Yin et al. found that moderate increases in Nrf2 (using mice heterogeneous for Keap1) significantly improves osteoblast formation and bone mass in male mice (Y. Yin et al., 2020). The latter study, unlike others, is one of the first to show that enhancement of Nrf2 may provide improved bone health in normal, healthy conditions.

Interestingly, Nrf2 signaling can be modulated via numerous mechanisms, which may hold promise for enhancing bone healing. Thalar et al. found that Sulforaphane, a natural inducer of Nrf2 signaling, enhances osteoblast differentiation, reduces apoptosis of osteoprogenitors, promotes apoptosis of pre-osteoclasts, and improves bone volume of both normal and ovariectomized mice (Thaler et al., 2016). This compound is currently being investigates as a potential to mitigate aging by activating Nrf2 in older adult (Northern Arizona University 2021). Unfortunately, few studies have set out to investigate the role of Nrf2 enhancing drugs on the direct effect on bone healing. In an elegant review by Kubo et al., there are other molecules and drugs, such as dimethyl fumarate, bardoxolone methyl, beta-agonists, VEGF, and others, that have positive regulatory effects on the Nrf2/ARE pathway and may improve fracture healing (Y. Kubo et al., 2019; J.-H. Kim et al., 2014; Takahata et al., 2009). Upregulating Nrf2 signaling may be a promising technique to increase bone-forming cells’ ability to suppress harmful intracellular ROS. However, these molecules have been largely understudied, and, while it is likely that increasing Nrf2/ARE signaling will improve bone healing in highly oxidative conditions, it is unclear if upregulating this pathway will improve normal physiological fracture repair. There is a great need for future research to investigate the direct role of Nrf2 in each phase of fracture healing and determine clinical situations in which Nrf2 modulation could provide beneficial outcomes.

## 5 Engineering Approaches to Modulate Reactive Oxygen Species in Fracture Healing

Bone tissue engineering (BTE) remains a relatively new yet promising alternative to the use of bone grafts in the treatment of bone disorders and injuries. BTE provides an unlimited supply of grafting resources and prevents disease transmission. Combinations of scaffolds, cells, and specific chemical and physical stimuli are being developed to optimize bone repair, regeneration, and treatment outcomes. The four major components of a successful BTE treatment require a biocompatible scaffold, osteogenic cells, morphogenic signals, and sufficient vascularization (Amini et al., 2012). While most seem to highlight the importance of an appropriate physiological range of ROS for maximal bone healing effects, how to utilize ROS for improved BTE treatment still needs to be clarified.

### 5.1 Mesenchymal Stem Cell Preconditioning to Improve Bone Healing

The data for the use exogeneous antioxidants to improve fracture healing is promising. One potential use of cellular priming techniques is to “teach” cells to handle oxidative environments. Studies have found that by pre-treating in oxidative environments, MSCs upregulate ROS-scavenging enzymes that result in improved tissue healing. These primed MSCs could then be combined with bio-scaffolds to improve fracture healing.

#### 5.1.1 Hydrogen Peroxide Preconditioning

Kubo et al. found that by simply preconditioning with low-dose hydrogen peroxide (H_2_O_2_), bone marrow-derived MSCs (BMSCs) had greater survival and enhanced neovascularization after being implanted into ischemic hindlimbs of mice. This study found that after 14 days of implantation, many more H_2_O_2_-pretreated BMSCs were viable compared to the untreated group. Further, these preconditioned BMSCs were able to significantly upregulate VEGF expression in ECs after only 1 day of implantation (M. Kubo et al., 2007). More recent studies are finding that preconditioning with oxidative environments are cellular protective and lead to improved viability, likely by the upregulation of antiapoptotic and pro-survival pathways (Nouri et al., 2019; He et al., 2020). Guo et al. further found that 50 μM H_2_O_2_ is the optimal dose to maximize their proliferation, survival, and migration BMSCs (Guo et al., 2020). This pretreatment upregulated PI3K/Akt/mTOR pathway, as well as cyclin D1, SDF-1, and CXCR4/7 receptors. After implanting these pre-treated cells into full thickness wounds of mice, they noticed improved engraftment and survival compared to untreated BMSCs. Interestingly, these pretreatments also upregulate key ROS-scavenging enzymes such as heme oxygenase 1, catalase, NQO1, and SOD through the Nrf2 pathway (F. Zhang et al., 2019; Yuan et al., 2017). Thus, these preconditioned cells have an increasingly reductive environment, allowing them to better suppress harmful levels of *intracellular* ROS during the fracture healing process. Additionally, as described in Figure 3, preconditioning with ROS upregulates IL-8, FGF2, and VEGF, which may prime MSCs to accelerate the inflammatory phase of fracture healing without tampering with the physiologic levels of extracellular ROS needed for healing.

Curcumin (a dietary product with cellular protective effects) has also been shown to suppress H_2_O_2_-induced oxidative stress in BMSCs (Yagi et al., 2013). Wang et al. found that by preconditioning with curcumin, along with hypoxic conditions, BMSCs had significantly better survival, proliferative capabilities, and mitochondrial function via the upregulation of PCG-1a/SIRT3/Hif-1α signaling. These preconditioned BMSCs also accelerated cutaneous wound healing in a mice model(X. Wang et al., 2020). Due to its low solubility and high liver metabolism, curcumin has poor efficacy as an oral antioxidant (Safali et al., 2019). However, curcumin-coated biopolymers have shown promise. Bose et al. found that human osteoblasts in curcumin-coated biopolymers had significantly improved viability and morphological characteristics. They also found that curcumin-coated 3D-printed scaffolds showed improved bone formation and mineralization in loadbearing and non-loadbearing implants (Bose et al., 2018). These results show promise for using curcumin locally as an antioxidant to improve bone healing.

#### 5.1.2 Hypoxic Preconditioning

Pre-culturing MSCs in hypoxic conditions replicate these protective results as well. Andreeva et al. found that adipose-derived MSCs cultured in hypoxic conditions had an increase in ROS, but also had an increase in superoxide dismutase and Hif-1α (Andreeva et al., 2015). Taken together, it is reasonable to assume that hypoxic conditions can both increase the ROS needed to upregulate FGF2, VEGF, and IL-8, while also increasing the ROS-scavenging mechanisms to control ROS-induced cellular damage. Thus, hypoxic pretreatments may be a promising technique to prime MSCs for accelerated fracture healing. Other studies have confirmed that hypoxic pre-treated MSCs upregulate VEGF and FGF2, as well as upregulating platelet-derived growth factor (PDGF) and polarizing macrophages to an anti-inflammatory M2 phenotype (Fotia et al., 2015; Maruyama et al., 2020). Furthermore, in an elegant series of experiments, Liu et al. discovered that hypoxia preconditioning of MSCs increases exosomal miR-126, which promotes bone fracture healing (Liu et al., 2020, 12).

While limited, the current literature suggests that hypoxic MSCs have improved osteogenic potential. Yu et al. found that hypoxic MSCs had enhanced osteogenic differentiation *in vitro* and accelerated fracture repair *in vivo* by upregulating Hif-1α and STAT3 phosphorylation (Yu et al., 2019). Further, Ho et al. found that MSCs pretreated under hypoxic conditions and then formed into spheroids entrapped in alginate gels significantly improved healing of a critical-sized bone defect, compared to gels with untreated MSC spheroids (Ho et al., 2018). Their preconditioned MSC spheroids were also found to be more resistant to apoptosis. Finally, hypoxia preconditioned MSCs implanted into bone-like bio-scaffolds showed increase collagen deposition when transplanted into subcutaneous tissue of mice. These promising studies clearly suggest hypoxic preconditioned MSCs may be an effective method to improve fracture healing. However, there is a need for more studies that directly study how preconditioned MSCs can improve *in vivo* fracture healing (Maruyama et al., 2020).

There are numerous other compounds that have been explored to precondition MSCs for the oxidative fracture healing environment. Each of the endogenous antioxidants previously discussed could be used as pretreatments to be infused into bio-scaffolds. For example, Watanabe et al. pretreated BMSCs with N-acetyl cysteine (NAC) before incorporating them into a collagen sponge. These sponges were then placed in a critical-sized rat femur defect and found that the NAC pretreated group resulted in significantly increased new bone formation (Watanabe et al., 2018). While not yet tested in a bio-scaffold for fracture repair, sodium ascorbyl phosphate (a vitamin C derivative) has been proposed as a suitable molecule for coating biomaterials and has been shown to both strongly stimulate osteogenesis and scavenge ROS (Okajima et al., 2020). Further, astaxanthin has recently been shown to protect MSCs from oxidative stress by upregulating Nrf2 signaling (Mohammadi et al., 2021).

Additionally, He et al. pre-treated adipose-derived MSCs (ASCs) with salidroside and hypoxic conditions and found these cells were able to resist H_2_O_2_-induced cell death. These two pretreatments acted synergistically to give MSCs better resistance to ROS. Further, they found that these conditions upregulated autophagy and activated Akt, Erk1/2, and LC3. These pre-treated cells also had enhanced migration and survival in the presence of oxidative stress (He et al., 2020). These results suggest another pretreatment option to prime MSCs for skeletal tissue healing. However, these pretreatment regimens have not been incorporated in a fracture healing model.

### 5.2 Molecular Targeting of Reactive Oxygen Species to Improve Bone Healing

A newer, promising approach to suppressing intracellular ROS levels is by using RNA interference (RNAi) technology, such as short interfering RNAs (siRNA) or microRNAs (miRNA) (Cerqueni et al., 2021; L. L.; Wang and Burdick 2017). In short, siRNAs are small, non-coding RNAs designed to degrade specific mRNA sequences before they are translated into proteins. miRNAs are also non-coding RNA molecules that can interfere with signaling pathways; however, they have a much broader effect by influencing a wider range of mRNA targets and can regulate protein expression (Ahmadzada et al., 2018). By using viral vector delivery systems, siRNA or miRNA can be reliably inserted into BMSCs to target relevant ROS-producing or antioxidant pathways. For instance, siRNAs could be delivered to BMSCs to silence the expression of NOX2, potentially dampening harmful intracellular ROS, while not interfering with pro-angiogenic levels of extracellular ROS. However, off-target effects and potential mutagenesis is a major limitation of delivering siRNAs (Cerqueni et al., 2021). Therefore, alternative delivery options are needed.

Promisingly, hydrogel scaffolds have been shown to be a reliable platform to deliver RNAi particles (M. K. Nguyen et al., 2018; L. L. Wang and Burdick 2017). Effectively, hydrogels encapsulate the RNAi (which is likely housed in a nanoliposome) particle within a 3D matrix (Ahmadzada et al., 2018; Cerqueni et al., 2021). This method limits the RNAi delivery to only local cells involved in the healing response and limits systemic effects. In theory, bone-forming cells that integrate into the hydrogel would be transfected with a RNAi particle that would improve the intracellular reducing environment and improve bone mineralization and healing. However, as extensively reviewed above, there are many cells involved in bone healing and, currently, there is no way of preventing the RNAi from being delivered to ECs or immune cells, where ROS inhibition could be detrimental to bone healing. There is great opportunity for further research in this promising area.

### 5.3 Other Bioengineering Techniques that Modulate Reactive Oxygen Species for Bone Healing

Osteogenic differentiation of MSCs is influenced by the production of ROS. Yang *et al.* emphasizes the importance of removing overproduced ROS to promote osteogenesis and bone healing. Their study uses microfluidic technology to prepare fullerol-hydrogel microfluidic spheres (FMSs) created by fullerol nanocrystals injected into hydrogel microspheres that essentially work as stem cell carriers (J. Yang et al., 2021). *In vitro* validatory studies demonstrated that these antioxidant spheres protect BMSCs from oxidative damage by capturing both intracellular and extracellular ROS, in addition to promoting osteoblast differentiation via activation of FOXO1 signaling. When injected into rats with calvaria defects, the FMNs appear to be the reason for significantly increased bone formation (J. Yang et al., 2021). Varini et al. engineered scaffolds using cerium (III) and (IV)-containing mesoporous glass beads, which have been shown to have antioxidant properties (Varini et al., 2019). This group found that osteoblasts cultured in these scaffolds had dampened ROS, improved cell viability, and increased proliferation (Varini et al., 2019). This new biomaterial has not yet been tested in a fracture healing model, and the mechanism behind reduced ROS and increase osteoblast proliferation is unknown.

Other groups have engineered smart biomaterials that sense oxidative stress and release antioxidants when they are needed (Cerqueni et al., 2021; Q.; Xu et al., 2016). These biomaterials consist of a structural framework that can entrap antioxidant compounds, and possibly nanoliposomes with RNAi particles as discussed previously. When these biomaterials encounter oxidative stress, functional groups change their conformation and the polymer network degrades, releasing the housed antioxidants. Of interest to bone regeneration, the number of antioxidants compounds can be altered proportionally, allowing for scalability depending on defect size or application. These biomaterials have a wide range of applications; however, few studies have tested their ability to improve bone regeneration (Yao et al., 2019; Shafiq et al., 2021). Further, Wu et al. has shown that ROS-sensing hydrogels can be combined with structural polymers to improve cartilage regeneration (X. Wu et al., 2021). This hybrid biomaterial allows for ROS-sensing technology, without the degradation of the structural framework that is needed for musculoskeletal applications.

Rather than focusing on reducing ROS, others have acknowledged that an increase in ROS is necessary for osteogenesis, especially for the formation of blood vessels to adequately vascularize bone. Bai et al. demonstrated the use of low-level laser therapy (LLLT) in promoting coupled angiogenesis and osteogenesis by use of a BMSC and biphasic calcium phosphate graft implanted into mice and an endothelial and bone marrow stem cell coculture (Bai et al., 2021). Both *in vivo* and *in vitro* studies demonstrated a significant increase in angiogenesis and osteogenesis as well as upregulation of VEGF, TGF-β, and Hif-1α. Through further investigation, it was found that these effects were induced through the ROS/Hif-1α signaling pathway, emphasizing LLLT’s production of ROS as the key feature in promoting angiogenesis and osteogenesis. While their results highlight the increase in ROS being beneficial to angiogenesis and osteogenesis, the study emphasizes a suitable window for osteogenic differentiation, with higher ROS levels damaging osteogenic differentiation at later timepoints (Bai et al., 2021).

Other studies have investigated the use of nanovibration and electrical stimulation (EStim) to produce physiological levels of ROS (Díaz-Vegas et al., 2015; Leppik et al., 2020; Orapiriyakul et al., 2020). Using a nanovibrational bioreactor, Orapiriyakul *et al.* investigated the effects of vibrational amplitude on osteogenesis in MSCs and the development of tissue engineered MSC-laden scaffold (Orapiriyakul et al., 2020). The increase in osteogenesis with an increased amplitude is found to be attributed to changes in adhesion, tension, and ion channel regulation, along with activation of energetic metabolic pathways that result in production of ROS and inflammation. Specifically, they looked at the increase in ROS induced by nanovibration and concluded that low-level ROS production and inflammation are rather byproducts of successful osteogenesis rather than drivers of the process, and inhibition of ROS results in small effects on osteogenesis (Orapiriyakul et al., 2020). While their conclusions are valid, this study did not investigate ROS levels on the magnitude present during fracture healing. Of course, the osteogenic differentiation process will increase levels of ROS due to the increase in oxidative phosphorylation and inhibiting this level of intracellular ROS (via NAC in this study) will likely not affect osteogenic differentiation. As described extensively above, the fracture healing environment has high levels of extracellular ROS and signaling molecules (Figure 3) that can further increase intracellular ROS to damaging levels. That said, this very new nanovibrational technology may have powerful applications in the future. Given that the various vibrational amplitudes can be transferred to collagen scaffolds, there may be an interesting opportunity to design a biofeedback control system that regulates ROS production depending on extracellular ROS and signaling molecules. Further, studies need to be done to investigate if nanovibration can also activate ROS-scavenging pathways, or if their activation is merely a byproduct of the increased ROS levels.

Additionally, a review on the use of EStim in bone tissue engineering shows increases in bone healing and regeneration via numerous cell response mechanisms, notably including controlled induction of ROS at an appropriate physiological level. This review highlights the numerous *in vitro* and *in vivo* experimentation surrounding EStim’s osteogenic benefit and future potential as a bone tissue engineering therapy (Leppik et al., 2020). EStim has shown to increase ROS via a ATP/PKC/NOX2 signaling cascade in skeletal muscle (Díaz-Vegas et al., 2015). Thus, much like the nanovibrational study, EStim may induce physiologic levels of ROS that increase osteogenic differentiation via stimulation of MAPK pathways (Díaz-Vegas et al., 2015; Leppik et al., 2020). Overwhelmingly, more studies are needed to test these technologies on bone healing in environments of high oxidative stress.

## 6 Current Challenges, and Future Perspectives

Our understanding of the effects of ROS on cellular processes has been more firmly established through cell biology research in the past several decades, but the therapeutic potential of how to utilize ROS is less agreed upon in the literature. We have outlined the current state of the limited research on how ROS are involved in the early stages of fracture healing, which consists of a range of approaches and some contradictory findings. Undoubtedly, ROS are necessary during the early phases of fracture healing. Thus, dampening these levels could interfere with normal, healthy skeletal tissue healing. Conversely, pathologically high levels of ROS can be damaging to bone healing cells and interfere with important signaling pathways in osteogenesis.

Numerous antioxidants have been explored to decrease ROS levels during skeletal tissue healing, with the hypothesis that they would improve results. However, the results are mixed and vary depending on a multitude of factors. As reviewed, oral antioxidants, such as vitamin C, vitamin E ( α -tocopherol), and NAC are promising; however, their benefits seem to be the most pronounced in high-ROS disease states, such as osteoporosis, diabetes, and rheumatoid arthritis. The effect of these antioxidants on normal fracture healing are contradictory. These mixed results are most likely due to the inhibition of healthy (necessary) levels of ROS. Further, it is reasonable to conclude that in larger fractures, an exogenous antioxidant will prohibit necessary levels of ROS to aid in neovascularization during fracture repair. Whereas using an antioxidant in a stable fracture may improve fracture healing via improved bone cell function.

Although there does appear to be opportunities in combining drugs, scaffolds, and engineering principles to optimize ROS levels for improved outcomes, further studies need to be conducted to explore this potential. The mechanisms in which ROS affect cellular processes is complex, as discussed in detail above, and understanding how they specifically contribute to bone homeostasis and repair is vital to developing more directed research for therapeutic applications.

## Glossary

ALP: alkaline phosphatase
ARE: antioxidant response element
ASC: adipose-derived Stem Cell
AT: α-tocopherol
BMSC: bone marrow-derived mesenchymal stem cell
BPM: bone morphogenic protein
BTE: bone tissue engineering
Dll4: notch ligand delta-like 4
EC: endothelial cell
ECM: extracellular matrix
EStim: electrical stimulation
ETC: electron transport chain
FGF: fibroblast growth factor
FMS: fullerol-hydrogel microfluidic spheres
FOXO: forkhead box O family of transcription factors
Gpx: glutathione peroxidase
H_2_O_2_: hydrogen peroxide
Hif-1 *α*: hypoxia-inducible factor 1 *α*
IGF-1: insulin-like growth factor 1
IL-8: interleukin-8
LLLT: low-level laser therapy
miRNA: microRNA
MSC: mesenchymal stem cell
mtROS: mitochondrial-produced reactive oxygen species
NAC: N-acetyl cysteine
NF-κB: nuclear factor-kappa B
NOX: NADPH oxidase
Nrf2: nuclear factor erythroid 2-related factor-2
O_2_•: superoxide
•OH: hydroxyl radical
PDGF: platelet-derived growth factor
PI3K: phosphoinositide 3-kinase
PPAR: peroxisome proliferator activated receptor
PRX: peroxide redox proteins
RNAi: RNA interference
ROS: reactive oxygen species
siRNA: short interfering RNA
SOD: superoxide dismutase
Tcf/Lef: T-cell factor/lymphoid-enhancing factor
VEGF: vascular endothelial growth factor
